# A conceptual regeneration–maintenance decision aid for class II furcation involvement

**DOI:** 10.1007/s00784-026-07039-8

**Published:** 2026-07-18

**Authors:** Anete Vaškevica, Meltem Özdemir Kabalak, Jenna Kompuinen, Ilze Akota, Ulvi Kahraman Gürsoy, Johan Caspar Wohlfahrt

**Affiliations:** 1 Riga Stradiņš University, Riga, Latvia; 2 Department of Periodontology, Institute of Dentistry, University of Turku, Turku, Finland; 3 Department of Periodontology, RSU Institute of Stomatology, Riga, Latvia; 4 Department of Periodontology, Faculty of Dentistry, Hacettepe University, Ankara, Turkiye Turkey; 5 Department of Periodontology, Faculty of Dentistry, University of Oslo, Oslo, Norway

**Keywords:** Furcation involvement, Periodontal regeneration, Clinical decision-making, Prognosis, Supportive periodontal therapy

## Abstract

**Objective:**

To introduce a practical, evidence-based and prognosis-oriented conceptual decision aid for the management of Class II furcation involvement, integrating defect-specific regenerative potential with long-term maintenance feasibility.

**Materials and methods:**

A structured narrative review of the literature on Class II furcation involvement regeneration, prognostic factors, and long-term maintenance outcomes was conducted using PubMed, EBSCO, and Scopus databases. Variables with documented influence on regenerative outcomes or long-term tooth survival were extracted and grouped into two conceptual axes: a Morphological Regenerability Score, capturing defect anatomy, and a Maintenance Accessibility Score, capturing site-related and patient-related factors. Scoring ranges and thresholds were derived from the magnitude and consistency of reported effect sizes.

**Results:**

The Morphological Regenerability Score integrates five defect-related variables (vertical furcation involvement component, root divergence at crestal bone, furcation entrance width, defect location and root trunk length) with modifiers for mobility, cervical enamel projections, exposed furcation entrance, and enamel pearls, spanning − 5 to 9 points. The Maintenance Accessibility Score integrates seven maintenance-related variables (plaque control, supportive periodontal treatment compliance, smoking, diabetes control, site/operator accessibility, endodontic status and tissue phenotype), spanning 0 to 13 points. Midpoint thresholds (MRS ≥ 5; MAS ≥ 7) define four clinical quadrants guiding case selection between regeneration, staged treatment strategy, non-surgical or resective approaches, and extraction.

**Conclusions:**

The proposed framework consolidates heterogeneous evidence on Class II furcation involvement prognosis and proposed treatment strategies into a single transparent decision aid. Prospective validation is required before routine clinical adoption.

**Clinical relevance:**

This framework supports evidence-based, individualised decision-making for Class II furcation involvement based on defect regenerability and long-term maintenance, and highlights modifiable risk factors that should be addressed before attempting regenerative surgery.

**Supplementary Information:**

The online version contains supplementary material available at 10.1007/s00784-026-07039-8.

## Introduction

Furcation involvement (FI) is defined as the extension of periodontitis-induced alveolar bone resorption into the bifurcation or trifurcation areas of multirooted teeth, representing a critical anatomical complication in the progression of periodontal disease. The anatomy of the furcation, which depends on the tooth type, demarcates the progression of tissue destruction and treatment success [1]. Although FI is associated with a nearly twofold increase in the risk of tooth loss compared with non-furcation-involved molars [2], with risk increasing by furcation class [3], there is broad professional consensus that FI alone, in the absence of additional unfavourable factors, does not constitute an indication for tooth extraction. Contemporary treatment frameworks position extraction as the last option in a hierarchy that begins with non-surgical therapy and progresses through regenerative or resective approaches before extraction is considered [4]. This hierarchy is reinforced by the EFP S3 clinical practice guideline, which recommends periodontal regenerative therapy in selected Class II FI defects - specifically mandibular Class II and buccal maxillary Class II FI as the treatment of choice when the defect morphology permits [5]. The management of Class II FI defects however remains a significant challenge even in contemporary clinical periodontal therapy. While the prognosis of FI is influenced by a combination of anatomical and patient-related factors, with optimal supportive periodontal therapy (SPT), favourable survival rates can be achieved, and under certain conditions, regenerative surgery can reduce both the horizontal and vertical components of the FI defect [6, 7]. Despite the availability of several tooth-level prognostic systems, simultaneous integration of regenerative potential and maintenance feasibility at the level of an individual furcation defect is still missing. McGuire and Nunn’s landmark study reported 81% overall accuracy for their regression model in predicting prognosis categories versus actual outcomes after 5 and 8 years of SPT; however, when teeth with ‘good’ prognosis were excluded, accuracy of the prediction fell to 43% at 5 years and 35% at 8 years [8]. A limitation was that the model scored FI only by horizontal severity, without capturing the vertical bone loss. Miller et al. used Kaplan–Meier survival analysis to assign weighted scores to six prognostic factors, namely age, periodontal probing depth (PPD), mobility, FI, smoking, and molar type, achieving a Harrell’s C-index of 67.1% (95% CI: 49.7–82.6%). This scoring system for regenerative decision-making in Class II FI predicted overall molar survival rather than guiding furcation-specific treatment, and FI was scored only by the number of affected furcations per molar, without using an established classification to capture defect severity [9].

In 2007 a new system was proposed for assessing periodontal prognosis, which was based on the periodontal stability of the tooth rather than its survival. The authors introduced a four-category classification system qualitatively defined according to whether contributing factors could be controlled and periodontal stability achieved. As a conceptual proposal, FI grade was not incorporated as a distinct prognostic variable [10].

In a 2011 retrospective study, Graetz et al. evaluated 34 patients with aggressive and 34 patients with chronic periodontitis, who were enrolled in a SPT regimen and followed for 10 or more years. The authors grouped all teeth into two categories: ‘questionable’ (≥ 50 to < 70% bone loss) and ‘hopeless’ (≥ 70% bone loss). Although the study described long-term retention, it did not test the predictive accuracy of this system. From the perspective of furcation-related decision-making, FI grade was not included as a prognostic variable and outcomes were not stratified by furcation status. Consequently, furcated and non-furcated teeth were analysed within the same prognostic categories [11].

Nibali et al. in a 2017 retrospective study assessed the risk of tooth loss in 100 patients with chronic periodontitis followed for at least 5 years during supportive periodontal treatment. The authors proposed a tooth prognosis system based on a decision tree model incorporating four different categories of bone loss levels as well as dichotomous checks on FI, mobility, PPD, periapical status and restorability. The system was associated with tooth loss in multivariable analysis, although the four categories were combined into two for statistical testing and FI was treated as a threshold check rather than graded variable [12].

It was confirmed in a 2016 systematic review and meta-analysis by Nibali et al. that furcation involvement increases the risk of molar loss in supportive periodontal therapy with a follow up period up to 10–15 years, with risk rising in FI grades II and III [2]. A subsequent retrospective study by Nibali et al. showed that both horizontal and vertical furcation components are independently associated with molar loss in patients with chronic periodontitis undergoing regular supportive treatment visits [13]. Later it was reported that molar loss rates increase with FI grade in populations which do not receive regular periodontal care, underlining the importance of careful diagnosis, treatment planning and maintenance [14]. Consistent with this, the AAP Regeneration Workshop concluded that regenerative therapy is a viable treatment option for FI defects, with Class II defects representing a highly predictable scenario when adverse local and systemic factors are controlled and strict SPT regimen is maintained [15]. The EFP S3 Clinical Practice Guideline reinforces this concept. In molars with residual pockets after the first two steps of therapy, it recommends regenerative surgery for mandibular Class II FI and suggests regenerative surgery for maxillary buccal Class II FI [5].

In a 2020 recommendation paper, Rasperini et al. further advanced furcation-specific treatment planning by proposing decision trees for the management and regeneration of furcation-involved molars. The framework incorporated the horizontal and vertical components of FI, interproximal bone level, root anatomy, residual attachment of the roots, and oral hygiene accessibility, alongside soft tissue characteristics and patient-related factors such as compliance and smoking status. Although based on the available literature combined with the authors’ clinical experience and not prospectively validated, the article represented an important shift toward a multidimensional and individualized approach to FI management [4].

Taken together, these frameworks support the value of regenerative therapy for furcation-involved molars, but leave a space to integrate defect anatomy with patient-related maintenance feasibility in everyday chairside decision-making. To fill this gap between published recommendations and clinical decision-making, we propose a hypothetic two-axis matrix. Axis 1, the Morphologic Regenerability Score (MRS), captures anatomic features of the defect. Axis 2, the Maintenance Accessibility Score (MAS), captures patient and site related factors that determine long-term maintenance feasibility. Variables supported by stronger evidence are assigned wider scoring ranges (0–3), while those supported with weaker evidence receive narrower ranges (0–2). The framework is conceptual and not yet validated prospectively.

## Materials and methods

A literature search was conducted in PubMed, EBSCO, and Scopus between April 2025 and May 2026 to identify evidence relevant to the treatment, prognosis, and regenerative management of furcation involvement. Only English-language articles published between 1980 and 2026 were considered. Randomized controlled trials, controlled clinical trials, prospective and retrospective cohort studies, case series with at least ten furcation-involved teeth, and systematic reviews were eligible for inclusion. Animal studies and in vitro studies were excluded. Case reports and case series with fewer than ten patients were excluded, with the exception of pre-specified histological case reports providing mechanistic evidence of periodontal regeneration in furcation defects. Search terms combined the anatomical anchor (furcation, bifurcation, trifurcation, interradicular) with therapeutic and prognostic descriptors (guided tissue regeneration, bone graft, enamel matrix derivative, growth factors, open flap debridement, non-surgical periodontal therapy, resective therapy, supportive periodontal therapy, tooth survival, tooth loss, prognosis, furcation classification), combined using Boolean operators AND/OR. Hand-searching was performed across the reference lists of all included studies and relevant systematic reviews, supplemented by manual screening of key periodontal journals to retrieve foundational and recent studies not captured by the electronic search.

## Results

According to a multicenter prevalence analysis involving 10,351 patients with periodontitis and furcation lesions, the most frequently observed FI type at the tooth level was Class I (66.47%), whereas at the patient level, Class II FI was the most common (44.05%) [16]. Most teeth with Class I FI can be successfully managed with non-surgical periodontal therapy (NSPT) and regular SPT, whereas the treatment of Class II FI is more complex, with a tooth loss risk twice as high as that of Class I FI [2, 17, 18]. Beyond prevalence, longitudinal data confirm that furcation involvement is a progressive condition: in a multi-center cohort of 3,924 patients followed for a mean of 4.7 years, 57.1% of patients experienced worsening of furcation involvement in at least one tooth, with a median time-to-progression of 3.6 years and a per-grade hazard ratio of 3.05 [19]. Although furcation lesions beyond Class II generally demonstrate less favorable outcomes, their prevalence is significantly lower than that of Class II FI [16, 20]. In a study of 600 patients who were re-examined at an average of 22 years after their active periodontal treatment, 460 of 1,464 teeth with FI at baseline were lost during SPT. Notably, more than half of these losses (240 teeth) occurred in approximately one-sixth of the patients who exhibited the most pronounced deterioration of the disease [21]. Similarly, in a cohort of 100 patients maintained for 15 or more years following periodontal therapy, 94 of 163 teeth (56.9%) initially diagnosed with FI were lost. Importantly, tooth loss was strongly dependent on the individual response to therapy, with substantially higher loss rates observed in patients exhibiting a downhill or extreme downhill disease course rather than on initial treatment alone [22].

The literature presents a diverse array of surgical and non-surgical methods for treating Class II FI. It is important to note that not all Class II FI defects share similar anatomical characteristics, leading to different treatment options for Class A2, Class B2, and Class C2 FI defects in the upper and lower [4, 5, 23, 24]. As outlined above, although more advanced furcation lesions generally present greater treatment challenges, Class II FI is significantly more prevalent and therefore represents the condition most routinely encountered in periodontal therapy.

### Open flap debridement

In a meta-analysis including 11 studies with a minimum follow-up period of 6 months, OFD resulted in a mean horizontal clinical attachment gain of 0.96 mm and a vertical gain of 0.55 mm. Although these outcomes represented a clinically significant improvement, none of the included cases demonstrated complete furcation closure or a change in furcation grade [25]. When compared with NSPT, OFD did not demonstrate superior PPD reduction or clinical attachment gain in the treatment of FI; however, it was associated with greater gingival recession [26]. Compared with OFD, regenerative surgical approaches demonstrated superior outcomes in terms of furcation improvement, horizontal and vertical clinical attachment gain, and PPD reduction in predominantly Class II FI defects [27].

### Regenerative therapy

Surgical regenerative approaches, including guided tissue regeneration, application of EMD, and bone grafting procedures, have demonstrated predictable outcomes in terms of horizontal furcation closure and long-term stability, particularly in maxillary buccal and mandibular Class II defects [28–30]. Regenerative surgery of predominantly Class II FI defects may achieve furcation closure or conversion to Class I involvement in a substantial proportion of treated defects [27]. Consistent with these findings, regenerative surgery showed the lowest tooth-loss rate (13%) over a mean follow-up of approximately 9 years among the evaluated treatment modalities in a retrospective cohort study of Class II FI defects [31]. Histological studies in humans have confirmed that true periodontal regeneration can be achieved in Class II FI following regenerative procedures [32, 33].

Current EFP S3-level clinical practice guidelines support regenerative approaches for appropriately selected buccal Class II FI defects and propose the use of EMD or various biomaterials, either alone or in combination with resorbable membranes as treatment options [5]. In a 2020 Bayesian network meta-analysis by Jepsen et al., including 20 randomized clinical trials predominantly involving Class II furcation involvement (FI) defects, bone replacement grafts demonstrated the most favourable horizontal bone gain, whereas non-resorbable membranes combined with bone replacement grafts produced the greatest vertical clinical attachment gain and probing pocket depth reduction [27]. In cases of maxillary interproximal Class II FI, the EFP clinical guidelines do not endorse a single superior treatment modality [5]. In addition to challenges in accessing interproximal Class II FI, regenerative outcomes are further compromised by technical difficulties in barrier membrane adaptation and stabilization [34]. Regenerative therapy appears most predictable in Class II FI defects with favourable morphology. However, the outcomes of regenerative therapy are influenced by both systemic conditions and local anatomic factors [30]. Moreover, patient-related factors, such as smoking and inadequate plaque control, remain critical determinants of regenerative success and often account for variability in the reported outcomes [35].

### Resective therapy

Resective approaches, including tunnelling procedures, root resection, or hemisection, may also be considered in advanced cases of Class II FI. While they do not aim to regenerate lost attachment, these modalities improve access for SPT and facilitate plaque control, and have been associated with acceptable long-term survival when strict maintenance is provided [36]. Despite the heterogeneity of the available studies, Dommisch et al. concluded in their 2020 systematic review that resective surgical therapy yields survival rates comparable to those of NSPT and OFD in Class II and III FI defects, with consistently better outcomes for Class II than Class III defects [37]. A 2025 retrospective cohort by Eickholz et al. is consistent with this conclusion: 83% (10 of 12) teeth treated with resective furcation surgery and 67% (2 of 3) teeth treated with tunnelling method were retained over a mean follow-up of approximately nine years of SPT, although small subgroup sizes limit generalization about this modality [31].

According to the EFP S3 level clinical practice guideline, root separation, root resection, and tunnelling procedures are among the proposed treatment options for interproximal or multiple Class II furcation defects [5]. Resective approaches may be particularly relevant in cases where complete regeneration is unlikely, but improved access for long-term maintenance can be achieved. In such cases, long-term success appears to depend largely on maintenance accessibility and patient compliance [36].

### Non-surgical periodontal therapy

Various studies have emphasized that NSPT alone may be inadequate in maintaining long-term periodontal stability in teeth with Class II FI, and flap elevation may be needed to improve the visibility and accessibility of furcation areas. Nevertheless, successful non-surgical management of buccal Class II FI defects using ultrasonic instrumentation has been reported, although interproximal sites demonstrated less reduction in probing depth despite comparable attachment gains [38]. However, it has also been shown that access flap surgery did not improve clinical outcomes when compared to NSPT in buccal defects [39]. In a longitudinal study assessing the 5- and 10-year survival of molars with FI, Majzoub et al. showed that both NSPT alone and in combination with access flap surgery were clinically viable treatment options for molars with shallow horizontal and vertical furcation defects. More favourable outcomes were observed in Class A/B1 FI defects, whereas Class A/B2 FI defects appeared to respond less predictably to these treatment modalities [26]. However, a recent retrospective cohort study reported tooth loss across treatment subgroups for Class II FI defects over a mean follow-up of 108.6 ± 36.5 months: 15% with SPT and subgingival instrumentation alone, 31% with open flap debridement (OFD), 33% with tunnelling, 13% with regenerative surgery, and 17% with resective furcation surgery [31].

A variety of adjunctive approaches have been proposed to enhance the clinical effectiveness of NSPT in Class II FI defects. A 2023 systematic review by Chatzopoulos et al. which included randomized controlled trials with a follow-up period of at least 3 months, indicated that in FI defects, local adjuvants - alendronate, rosuvastatin, simvastatin, boric acid and tetracycline - used in combination with NSPT, improve clinical parameters in the short term over NSPT alone [40]. Additional furcation-specific trials, not included in this 2023 review, have evaluated locally delivered doxycycline gel during supportive periodontal therapy [41, 42] and aloe vera gel [43] as an adjunct in Class II FI defects, with limited and short-term outcome data. Other adjunctive agents, including photodynamic therapy, povidone-iodine, enamel matrix derivatives (EMD), and Nd laser therapy, have also been investigated as means of enhancing the efficacy of NSPT in the treatment of FI defects [44–46]. However, these short-term clinical trials have reported heterogeneous outcomes, resulting in inconclusive evidence regarding their effectiveness. Beyond these approaches, newer adjunctive strategies including probiotics, propolis, hyaluronic acid, and biomaterial-based agents such as chitosan have also attracted interest because of their potential anti-inflammatory, antimicrobial, or wound-healing properties however they have not been tested specifically in FI defects yet [47–52]. Chitosan-based devices have also been proposed for minimally invasive, repeated subgingival application, however, evidence in FI defects is lacking [51, 52]. Consequently, the available evidence remains heterogeneous and predominantly limited to short- to medium-term outcomes, and current clinical guidelines do not support the routine use of adjunctive therapies due to insufficient evidence.

## Framework construction

Following the review of the epidemiology, prognosis, and available treatment approaches for furcation involvement, a conceptual framework was developed to integrate morphologic and maintenance-related factors relevant to regenerative decision-making at the individual furcation level.

Each Class II FI defect is assessed on both Axis 1 and Axis 2. The Morphologic Regenerability Score (MRS) located on Axis 1 reflects the surgical regenerative potential of the tooth based on defect morphology. The Maintenance Accessibility Score (MAS) located on Axis 2 reflects what the patient and operator can sustain over time. Together, these two scores place the FI defect into one of four clinical quadrants, guiding the treatment recommendation. A distinctive feature of this matrix is that the width of each scoring range is informed by published effect sizes rather than assigned arbitrarily.

### Axis 1 - MRS

The MRS scores five anatomic variables. The vertical component of the defect presents the strongest available evidence on prognosis and is scored 0–3. Furcation entrance width, defect location, and root trunk length are scored 0–2, reflecting evidence that is moderate, indirect, or morphology-based [5, 15, 53–55]. Root divergence at the crest of bone (RDCB) is scored 0–1, matching the binary outcome contrast reported by Bowers et al. [56]. The maximum score is 9 (Table 1). Local modifiers (Table 2) are then subtracted in the presence of unfavourable conditions. While the final MRS can theoretically range from − 5 to 9, scores below 0 are clinically assigned a value of 0 for quadrant classification.

**Table 1:** Axis 1 - MRS weighted variables

Variable	Score 3	Score 2	Score 1	Score 0
Vertical component (0–3)	Subclass A (1–3 mm)	Subclass B (4–6 mm)	Subclass C (≥ 7 mm)	—
Root divergence at crestal bone, RDCB (0–1)	—	—	RDCB ≤ 3 mm	RDCB ≥ 4 mm
Furcation entrance width (0–2)	—	> 0.75 mm	0.5–0.75 mm	< 0.5 mm
Defect location (0–2)	—	Mandibular buccal/lingual	Maxillary buccal	Interproximal
Root trunk length (0–1)	—	—	Short or moderate (Type A or B)	Long (Type C)

**Table 2:** Axis 1 - Modifiers: tiered by evidence strength

Modifier (if present)	Penalty	Evidence tier
Tooth mobility Class 3	−2	Strong
Tooth mobility Class 2	−1	Strong
Cervical enamel projection (Grade II–III)	−1	Moderate
Clinically exposed furcation entrance	−1	Moderate
Enamel pearl at furcation	−1	Limited

### Rationale for MRS variable weighting

The vertical depth of the FI defect is a morphologic variable with consistent, multi-cohort outcome data linking it to tooth survival. Effect sizes are large and reproducible across surgical and prognostic studies. A four-step range (0–3) was selected for vertical defect component because the available evidence suggests at least three clinically meaningful gradations: shallow vertical defects with favourable outcomes, moderate defects with intermediate prognosis, and deep defects associated with a higher risk of treatment failure. Several authors have recommended assessing both horizontal and vertical components, noting that vertical depth shapes prognosis more strongly than any other anatomic feature [4, 6, 7, 57]. This variable therefore carries the widest scoring range in the matrix.

RDCB (0–1) determines how well a regenerative membrane or graft can be physically contained between the roots. Bowers et al. [56] reported that RDCB ≤ 3 mm achieved 93% complete furcation closure compared with 61% at RDCB ≥ 4 mm. In the proposed matrix, RDCB is scored on a 0–1 scale to reflect this binary outcome contrast directly: ≤3 mm is considered favourable (1), whereas ≥ 4 mm is considered unfavourable (0). As no outcome studies have stratified RDCB using additional thresholds, a wider scoring range is not currently supported by the available evidence. Limiroli et al. found that broader radiographic root divergence ratios were not significant predictors of regenerative outcomes, suggesting that functional containment at the defect level may be more clinically relevant than overall root morphology [58].

Inclusion of furcation entrance width (0–2) in the matrix is supported by morphologic evidence from Bower [53], Mukherjee et al. [59] and Ghishan et al. [60] demonstrating that a substantial proportion of furcation entrances are inaccessible to standard periodontal instruments, with 58% reported to be narrower than a standard curette blade [53] and 48.60% did not allow instrument engagement with the FI defect [59] and direct three-dimensional digital analysis of 240 extracted posterior teeth confirming that furcation entrance dimensions and inter-radicular root concavities are consistently below the working width of standard curette blades (0.75–1.10 mm) [60]. However, in contrast to expectations based on instrument accessibility alone, a 2004 study by Horwitz et al. demonstrated only a borderline association (*p* = 0.054), with wider entrances associated with less favourable healing outcomes, possibly because excessively wide openings may impair clot stability [61]. Given the strong morphologic rationale but limited and inconsistent outcome evidence, a moderate 0–2 scoring range was selected.

Defect location (0–2) is supported as a prognostic factor for regeneration by two randomized controlled trials from Pontoriero and Lindhe, and Pontoriero et al. which established a hierarchy whereby mandibular FI defects demonstrate the most favourable regenerative outcomes, maxillary buccal FI defects show intermediate outcomes, and interproximal FI defects respond least favourably [62, 63]. The 2020 Bayesian network meta-analysis of Jepsen et al. confirms the overall regenerative benefit at the defect types these randomized controlled trials studied [27].

Both the AAP Regeneration Workshop and the EFP S3 guideline reflect this hierarchy in their recommendations: mandibular Class II and buccal maxillary Class II FI defects are recommended for regenerative therapy, while interproximal sites are not [5, 15].

Root trunk length (0–1) influences both the anatomical space available for periodontal regeneration and the geometry of membrane adaptation. The Type A/B/C root trunk classification proposed by Hou et al. [55] is used in the present matrix to categorize root trunk dimensions relative to bone loss. Horwitz et al. [61] identified the furcation fornix - cementoenamel junction line distance, a radiographic measure of root trunk length, as a significant prognostic factor for horizontal attachment gain in Class II furcation defects treated with barrier membrane regeneration. Long root trunks were independently identified as a negative prognostic factor for healing in Class II furcation defects. As this is the only regenerative outcome study evaluating root trunk length in Class II FI defects, and because the variable was analysed continuously rather than categorically, the present matrix uses a binary 0–1 score: long root trunks (Type C) score 0, whereas shorter root trunks (A and B) score 1.

### Rationale for modifiers

Modifiers are subtracted from the MRS when unfavourable local conditions are present. The penalty magnitude reflects evidence strength.

Direct regenerative outcome data for mobility in Class II FI defects are lacking; therefore, the present rationale draws on adjacent periodontal evidence. Fleszar et al. demonstrated that mobile teeth gained less attachment following periodontal therapy than firm teeth of comparable disease severity [64]. Cortellini et al. reproduced this association in intrabony defects, identifying baseline mobility as a significant predictor of reduced CAL gain, although furcation defects were excluded [65]. In contrast, Trejo and Weltman found no significant differences in PD reduction or CAL gain between teeth with Class 1–2 mobility and firm teeth [66]. The only study directly linking mobility and furcation involvement was the large retrospective 2026 analysis by Chatzopoulos and Wolff which reported baseline mobility and furcation involvement as independent predictors of tooth loss, with adjusted odds ratios of 1.95 for Class 2 and 3.99 for Class 3 mobility [67]. Accordingly, the matrix assigns − 2 for Class 3 mobility, −1 for Class 2 mobility, and no penalty for Class 1 mobility. Mobility was retained as a modifier rather than a primary variable because it is partially reversible following nonsurgical therapy: in another recent cohort of 489 teeth, 71.2% of Class 1 and 42.2% of Class 2 mobile teeth returned to Class 0 stability twelve months after SRP, while persistent mobility was independently predicted by deeper probing depth, furcation involvement, smoking, and diabetes [68].

Cervical enamel projections (CEP) Grade II–III and clinically exposed furcation entrances each carry a −1 penalty. Hou and Tsai demonstrated significantly worse probing depth, attachment loss, plaque accumulation, and gingival inflammation in furcation-involved mandibular molars with CEPs or intermediate bifurcational ridges compared with sites without these [69].

Enamel pearls also receive a −1 penalty. Romeo et al. 2011 case report described multiple enamel pearls at maxillary molar furcations that prevented connective tissue attachment and contributed to localized periodontal breakdown, with two affected molars ultimately requiring extraction despite non-surgical therapy [70]. As the evidence for enamel pearls is currently limited, this penalty should be interpreted cautiously.

### Axis 2 - MAS

The MAS scores seven patient-, site-, and behaviour-related variables that influence long-term periodontal stability and the feasibility of SPT in furcation-involved teeth (Table 3). Unlike the morphology-driven MRS, the MAS captures factors that determine whether treatment outcomes can be maintained over time. Six variables, plaque control, SPT compliance, smoking, diabetes control, site/operator accessibility and endodontic status are scored from 0 to 2, whereas tissue phenotype/mucogingival conditions is scored from 0 to 1, resulting in a maximum possible score of 13 points. No additional modifiers are used in the MAS, as the variables themselves function as continuous determinants of maintenance predictability.

**Table 3:** Axis 2 - MAS weighted variables

Variable	Favourable (2)	Moderate (1)	Unfavourable (0)
Plaque control	Consistently good	Variable	Poor
Compliance to SPT	Regular	Irregular	Poor
Smoking	Non-smoker	Smokers consuming < 10 cigarettes/day	Smokers consuming ≥ 10 cigarettes/day
Diabetes Control	No diabetes	Controlled Diabetes (HbA1c < 7.0%)	Uncontrolled Diabetes (HbA1c ≥ 7.0%)
Site/operator accessibility	Favourable access	Moderate access	Limited access
Endodontic status	Vital pulp	Adequate root canal filling (RCF) with no periapical (PA) pathology	Inadequate RCF, PA pathology, or evidence of vertical root fracture
Tissue Phenotype/Mucogingival conditions	-	Thick/adequate Keratinized tissue (KT)	Thin/inadequate KT

### Rationale for MAS variable weighting

Plaque control (0–2) was included in the MAS because effective plaque control is considered essential for long-term periodontal stability and maintenance of treatment outcomes. In a 10-year longitudinal cohort study, Eickholz et al. identified mean plaque index during SPT as a significant risk factor for both tooth loss (*p* < 0.0001) and deterioration in periodontal status (*p* = 0.011), alongside irregular SPT attendance. This association is particularly relevant in molars with FI, where anatomical complexity compromises access for both patient-performed oral hygiene and professional debridement [71]. Dannewitz et al. highlighted this limited accessibility as a contributing factor to the disproportionately high tooth loss observed in furcation-involved teeth [72]. A three-level scale was selected to distinguish consistently good (2), variable (1), and persistently poor (0) plaque control, based on longitudinal clinical assessment rather than a fixed numerical threshold, since plaque indices vary across studies.

Compliance with SPT (0–2) was included because of its well-established association with tooth retention and long-term periodontal stability. In addition to the findings reported by Eickholz et al., other long-term observational studies have demonstrated substantially greater tooth loss and less favourable disease progression among patients with irregular maintenance attendance [71, 73, 74]. In a recent multicentre retrospective cohort study, regular SPT (≥ 2 visits/year) was associated with an approximately 50% lower adjusted hazard of progression in both Class I and Class II FI defects [75]. Another recent cohort including only teeth with Class II FI also identified regular SPT as a factor associated with improved long-term tooth retention [31]. A three-level scale was selected to reflect clinically meaningful differences in maintenance adherence patterns. Based on the available evidence, two or more SPT visits per year were considered to represent regular compliance (2), whereas irregular (1) and poor (0) attendance received lower scores according to expected maintenance predictability.

Smoking (0–2) was included because smoking has been associated with impaired periodontal healing and increased risk of tooth loss [76]. In a long-term retrospective cohort of periodontally treated molars, smokers showed a significantly higher risk of molar loss during SPT than non-smokers [72]. The three-level scale was aligned with the 2017 periodontal and peri-implant diseases classification framework, distinguishing non-smokers (2), smokers consuming fewer than 10 cigarettes/day (1), and smokers consuming 10 or more cigarettes/day (0) [77]. Although former smoking history was considered clinically relevant, it was not incorporated as a separate category because cumulative tobacco exposure and smoking cessation duration may vary substantially among former smokers, resulting in heterogeneous risk profiles [78].

Diabetes control (0–2) was included because inadequate glycemic control may negatively affect periodontal healing and long-term periodontal stability [79]. In a long-term retrospective cohort of periodontally treated molars, diabetes mellitus was associated with an increased risk of molar loss during SPT, although the number of diabetic patients was limited [72]. The three-level scale was aligned with the 2017 periodontal and peri-implant diseases classification framework, distinguishing patients without diabetes (2), patients with diabetes and HbA1c < 7.0% (1), and patients with diabetes and HbA1c ≥ 7.0% (0) [77]. However, a 2021 systematic review on tooth loss during long-term periodontal maintenance by Carvalho et al. did not consistently confirm baseline smoking and diabetes as predictors in meta-regression analyses; therefore, these variables were interpreted as maintenance-related risk modifiers rather than deterministic predictors of tooth loss [80].

Site/operator accessibility (0–2) was included because anatomical access may influence both the effectiveness of debridement procedures and the ability to maintain plaque control during supportive care. Buccal Class II FI defects were considered more favourable because they generally provide better access for instrumentation and maintenance [38]. Interproximal Class II FI defects were assigned an intermediate score due to restricted access by adjacent teeth and the associated challenges in plaque control and regenerative procedures [15, 38]. Multiple Class II FI defects were considered less favourable because recent cohort data showed that multiple Class II furcations per tooth were associated with poorer long-term prognosis and increased tooth loss risk [31]. Accordingly, a three-level scale was selected to distinguish favourable buccal-only furcation access (2), moderate interproximal access (1), and multiple Class II FI defects where access is limited (0) according to expected maintenance accessibility.

Endodontic status (0–2) was included because it is supported by evidence from several studies. In a SPT cohort of 1015 molars with a follow up period of at least 10 years (mean 13.2 ± 2.8), Dannewitz et al. [81] reported a multivariable Cox hazard ratio of 2.98 (*p* < 0.001) for endodontically treated molars, independent of FI, bone loss, and host factors. The endodontic subgroup of the same cohort [82] that looked at 188 RCF molars confirmed these findings (HR 2.98, 95% CI 1.74–5.1, *p* < 0.001). Molars with PAI scores 1–2 were grouped as ‘clinically healthy’. Of molars with PAI 4–5 46.2% were lost compared with the clinically healthy group (*p* = 0.06). Filling homogeneity was independently prognostic - retention reached 75% at homogeneity score 2 and 54% at score 3. Under- or overfilled root canal fillings were significantly more often associated with elevated PAI scores (*p* = 0.009). In this cohort patient-level factors (sex, compliance, smoking) did not affect retention among RCF molars, supporting endodontic status as a tooth-level variable. In the Class II furcation-specific cohort of Eickholz et al. 2025, endodontic treatment carried an odds ratio of 8.107 (37% vs. 16% molar loss) [31].

Scoring is therefore: 2 - vital pulp, no RCF, no PA pathology; 1 - adequate, well-condensed RCF with PAI 1–2 and no PA radiolucency; 0 - inadequate/under- or overfilled RCF, PAI ≥ 3 (especially 4–5), PA pathology, vertical root fracture, or post/core failure.

Tissue phenotype and mucogingival conditions (0–1) were included because they may influence plaque control, maintenance accessibility, and the feasibility of regenerative procedures in furcation-involved teeth. Adequate keratinized tissue width and vestibular depth may facilitate self-performed plaque control and soft tissue management during regenerative therapy, whereas thin or inadequate KT may increase technical complexity [4, 83] According to the AAP Regeneration Workshop consensus report on periodontal soft tissue non-root coverage procedures, a minimum of 2 mm of KT, including at least 1 mm of attached gingiva, may be beneficial for preventing attachment loss in patients with suboptimal plaque [83]. In addition, evidence suggests that thin gingival phenotypes may be associated with a greater tendency for gingival recession than thicker phenotypes, although specific prognostic thresholds remain uncertain [84]. Since direct evidence in Class II FI defects remains limited, a two-level scale was selected to reflect clinically relevant differences in soft tissue conditions rather than a validated prognostic threshold: thick/adequate KT (1) and thin/inadequate KT (0).

### Quadrant assignment and clinical interpretation

**Fig. 1:**
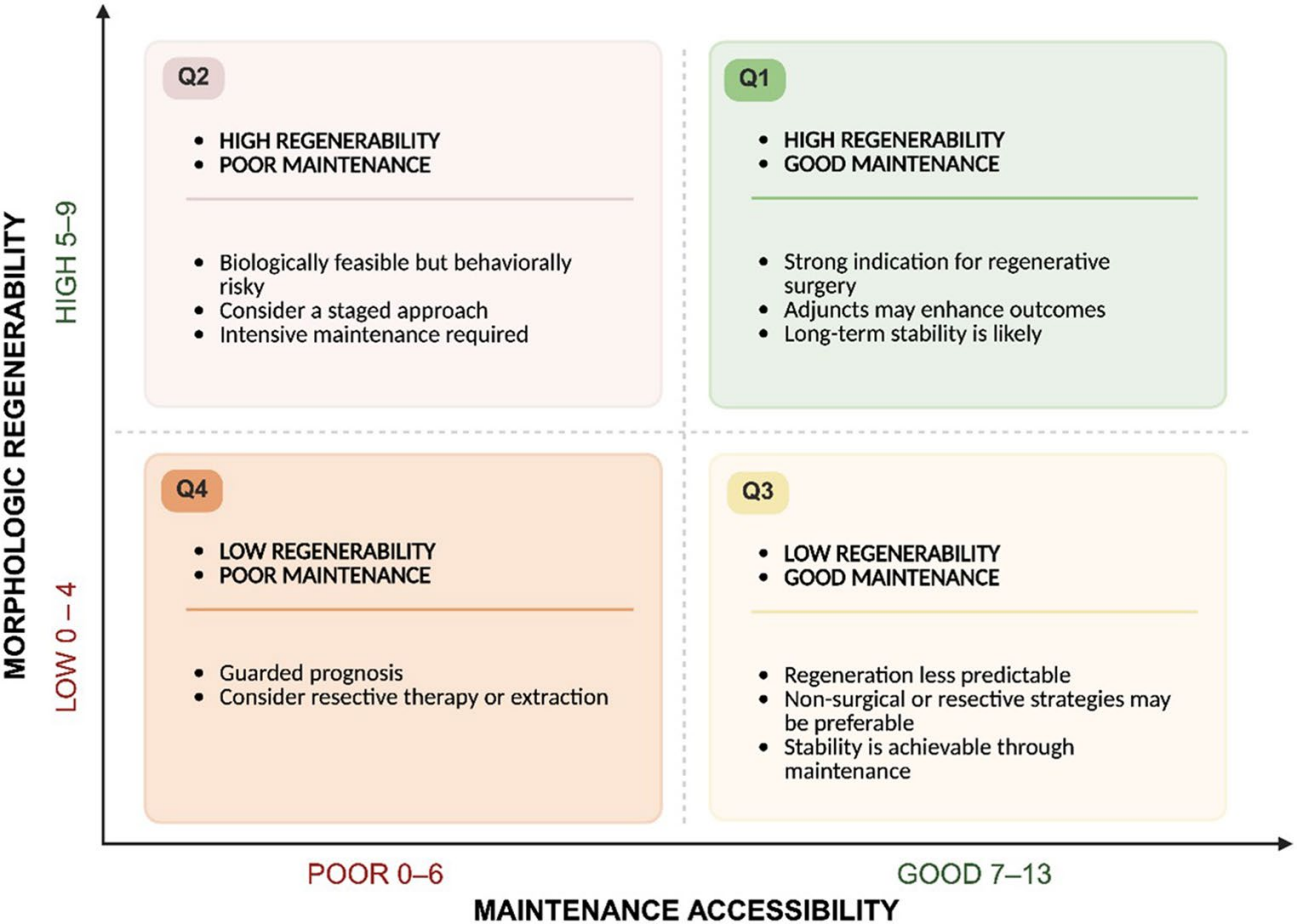
Two-axis decision matrix stratifying Class II furcation involvement defects by morphologic regenerability and maintenance accessibility to guide prognosis-driven treatment selection

The MRS and MAS scores together place each FI defect in one of four quadrants (Fig. 1). The threshold for each axis is set near the midpoint of its positive range: MRS ≥ 5 indicates high regenerability (on a scale of − 5 to 9, where negative scores denote non-regenerable defects); MAS ≥ 7 (out of 13) indicates high maintainability. These thresholds are starting points and should be tested in prospective cohorts.

Quadrant 1 (MRS ≥ 5, MAS ≥ 7 - High/High): The best candidates for regenerative surgery. Both the defect anatomy and patient conditions support a good outcome. The EFP S3 guideline recommends regeneration for mandibular Class II and buccal maxillary Class II FI as standard of care [5].

Quadrant 2 (MRS ≥ 5, MAS < 7 - High/Low): Favourable defect anatomy but compromised patient conditions. The defect could respond well to regeneration, but the risk of relapse without adequate maintenance is high. Modifiable MAS factors, particularly smoking and SPT compliance, should be addressed first.

Quadrant 3 (MRS < 5, MAS ≥ 7 - Low/High): Limited regenerative potential but good maintenance conditions. The patient can sustain the tooth well. Non-surgical strategies with or without local adjuncts, open flap debridement, or resective approaches are more appropriate than regeneration.

Quadrant 4 (MRS < 5, MAS < 7 - Low/Low): The highest-risk scenario. Both the anatomy and patient conditions are unfavourable. Extraction or maintenance-focused management may need to be weighed against the overall treatment plan and the strategic value of the tooth.

## Conclusions

To our knowledge, study that has simultaneously examined all five MRS or all seven MAS variables in a single multivariable model for Class II FI regeneration is missing in the literature. The effect sizes used here come from separate studies with different populations, follow-up periods, and analytical methods. The translation of individual HRs and ORs into ordinal scoring ranges is a structured approximation, not a statistically derived weighting.

One of the limitations of this proposed decision aid is that no outcome data stratify RDCB beyond the ≤ 3 mm and ≥ 4 mm contrast reported by Bowers et al. 2003 [56]. RDCB values within the intermediate 3–4 mm range fall outside the current scoring and require future evidence for resolution.

The modifiers for CEP (Grade II–III) and clinically exposed furcation entrance have limited supporting scientific evidence. The modifier for enamel pearls is supported by mechanistic reasoning and clinical case observation rather than RCT outcome data therefore its penalty should be treated as provisional until controlled studies are available.

The midpoint thresholds (MRS ≥ 5 on a scale of − 5 to 9, where negative scores denote non-regenerable defects; MAS ≥ 7 out of 13) are pragmatic starting points and prospective validation is needed.

Finally, this framework should be prospectively validated. Sensitivity analyses exploring alternative weighting schemes and the potential integration of machine learning for personalised threshold calibration represent important future directions.

## Supplementary Information

Below is the link to the electronic supplementary material.

ESM1 Supplementary file 1 (DOCX 32.0 KB)

## Data Availability

No datasets were generated or analysed during the current study.
