# Supplementary material for: A conceptual regeneration–maintenance decision aid for class II furcation involvement
Source: Clin Oral Investig. 2026 Jul 18;30(8):346. doi: 10.1007/s00784-026-07039-8 (PMC13380591; doi:10.1007/s00784-026-07039-8)
Supplement: Supplementary file 1 [file 784_2026_7039_MOESM1_ESM.docx]

Supplementary material

## Illustrative case: Class II mandibular lingual furcation

Case description: patient is a non-smoking female with no relevant medical history and a mandibular molar with Class II lingual furcation involvement. Tooth is vital with no endodontic treatment and no periapical pathology.

Site description:  FI with vertical subclass A (1–3 mm), root divergence at crestal bone (RDCB) ≤3 mm on periapical radiograph, furcation entrance width is 0.5–0.75 mm, root trunk Type A/B. The tooth has Class 2 mobility and thin/inadequate keratinised tissue.

Behavioural description: This patient has shown variable plaque control, however is compliant with supportive periodontal therapy (SPT) regimen.

Application of the Framework. 

Step 1 — Score Axis 1 (MRS). Apply Table 1 (anatomic variables) and then subtract any applicable modifiers from Table 2.

| MRS variable | Case finding | Score | Max |
| --- | --- | --- | --- |
| Vertical component (0–3) | Subclass A (1–3 mm) | 3 | 3 |
| Root divergence at crestal bone, RDCB (0–1) | ≤3 mm | 1 | 1 |
| Furcation entrance width (0–2) | 0.5–0.75 mm | 1 | 2 |
| Defect location (0–2) | Mandibular lingual | 2 | 2 |
| Root trunk length (0–1) | Type A/B (short/moderate) | 1 | 1 |
| MRS subtotal (Table 1) |  | 8 | 9 |
| Modifier — Mobility Class 2 (Strong evidence tier) | Present | −1 |  |
| MRS total |  | 7 | 9 |

Step 2 — Score Axis 2 (MAS). Apply Table 3 across all seven patient-, site-, and behaviour-related variables.

| MAS variable | Case finding | Score | Max |
| --- | --- | --- | --- |
| Plaque control (0–2) | Variable | 1 | 2 |
| Compliance to SPT (0–2) | Regular | 2 | 2 |
| Smoking (0–2) | Non-smoker | 2 | 2 |
| Diabetes control (0–2) | No diabetes | 2 | 2 |
| Site/operator accessibility (0–2) | Mandibular buccal/lingual (favourable) | 2 | 2 |
| Endodontic status (0–2) | Vital pulp | 2 | 2 |
| Tissue phenotype / mucogingival conditions (0–1) | Thin / inadequate KT | 0 | 1 |
| MAS total |  | 11 | 13 |

Step 3 - Quadrant assignment. Apply midpoint thresholds (MRS ≥5; MAS ≥7). This results in Quadrant 1 (High / High) assignment.

- MRS = 7 (≥5 - High regenerability)

- MAS = 11 (≥7 - High maintainability)

Step 4 — Treatment recommendation. Per the Quadrant Assignment and Clinical Interpretation subsection, Quadrant 1 defects are the best candidates for regenerative surgery, consistent with the EFP S3 recommendation of regeneration as standard of care for mandibular Class II FI (Sanz et al. 2020). Mucogingival deficiency (thin/inadequate KT, MAS = 0 for this variable) should be addressed before or simultaneously with the regenerative procedure to optimise soft tissue conditions for primary wound closure and long-term maintenance.
